# CaMKK2 Suppresses Muscle Regeneration through the Inhibition of Myoblast Proliferation and Differentiation

**DOI:** 10.3390/ijms17101695

**Published:** 2016-10-24

**Authors:** Cheng Ye, Duo Zhang, Lei Zhao, Yan Li, Xiaohan Yao, Hui Wang, Shengjie Zhang, Wei Liu, Hongchao Cao, Shuxian Yu, Yucheng Wang, Jingjing Jiang, Hui Wang, Xihua Li, Hao Ying

**Affiliations:** 1 Key Laboratory of Food Safety Research, Institute for Nutritional Sciences, Shanghai Institutes for Biological Sciences, Chinese Academy of Sciences, University of Chinese Academy of Sciences, Shanghai 200031, China; yecheng@sibs.ac.cn (C.Y.); zhangduo@sibs.ac.cn (D.Z.); liyan02@sibs.ac.cn (Y.L.); xhyao@sibs.ac.cn (X.Y.); wanghui01@sibs.ac.cn (H.W.); zhangshengjie@sibs.ac.cn (S.Z.); liuwei01@sibs.ac.cn (W.L.); hccao@sibs.ac.cn (H.C.); sxyu@sibs.ac.cn (S.Y.); huiwang@sibs.ac.cn (H.W.); 2 Department of Neuromuscular Disease, Children’s Hospital of Fudan University, Shanghai 201102, China; zhaolei1117@126.com (L.Z.); xihual@vip.sina.com (X.L.); 3 Shanghai Xuhui Central Hospital, Shanghai Clinical Center, Chinese Academy of Sciences, Shanghai 200031, China; candywang666@hotmail.com; 4 Department of Endocrinology and Metabolism, Zhongshan Hospital, Fudan University, Shanghai 200031, China; superseraph@126.com; 5 Key Laboratory of Food Safety Risk Assessment, Ministry of Health, Beijing 100021, China

**Keywords:** CaMKK2, muscle regeneration, proliferation, differentiation, AMPK

## Abstract

Skeletal muscle has a major role in locomotion and muscle disorders are associated with poor regenerative efficiency. Therefore, a deeper understanding of muscle regeneration is needed to provide a new insight for new therapies. CaMKK2 plays a role in the calcium/calmodulin-dependent kinase cascade; however, its role in skeletal muscle remains unknown. Here, we found that CaMKK2 expression levels were altered under physiological and pathological conditions including postnatal myogensis, freeze or cardiotoxin-induced muscle regeneration, and Duchenne muscular dystrophy. Overexpression of CaMKK2 suppressed C2C12 myoblast proliferation and differentiation, while inhibition of CaMKK2 had opposite effect. We also found that CaMKK2 is able to activate AMPK in C2C12 myocytes. Inhibition of AMPK could attenuate the effect of CaMKK2 overexpression, while AMPK agonist could abrogate the effect of CaMKK2 knockdown on C2C12 cell differentiation and proliferation. These results suggest that CaMKK2 functions as an AMPK kinase in muscle cells and AMPK mediates the effect of CaMKK2 on myoblast proliferation and differentiation. Our data also indicate that CaMKK2 might inhibit myoblast proliferation through AMPK-mediated cell cycle arrest by inducing cdc2-Tyr15 phosphorylation and repress differentiation through affecting PGC1α transcription. Lastly, we show that overexpressing CaMKK2 in the muscle of mice via electroporation impaired the muscle regeneration during freeze-induced injury, indicating that CaMKK2 could serve as a potential target to treat patients with muscle injury or myopathies. Together, our study reveals a new role for CaMKK2 as a negative regulator of myoblast differentiation and proliferation and sheds new light on the molecular regulation of muscle regeneration.

## 1. Introduction

Skeletal muscle is an important part of the animal body, and represents nearly half of the total body mass [1]. The skeletal muscles coordinate body movements through their attachment to the skeleton. A skeletal muscle is composed of many bundles of myofibers, while a single myofiber is derived from the fusion of numerous myoblasts and contains many nuclei [2]. Muscle development determines the muscle fiber number, muscle mass, muscle fiber type and some muscle pathological changes. The process of muscle development can be divided into several major stages in vertebrate embryos, such as myogenic precursor proliferation and differentiation into myoblasts, myoblast proliferation, determination, differentiation and fusion into myotubes [3]. Among these development stages, differentiation is an important process, which determines muscle cell fate and final muscle formation, and it is well studied in embryonic myogenesis due to C2C12 mouse myoblast model [3]. However, postnatal, muscle growth depends on remodeling pre-existing fibers, and muscle differentiation only exists in skeletal muscle regeneration after injury. The main stem cells contributing to muscle fiber repair are satellite cells, which maintain in a quiescent state in healthy muscle. Upon injury, the satellite cells are activated and proliferate to muscle progenitor cell and differentiate to form myofibers.

CaMKK2 is an enzyme, which plays a role in the calcium/calmodulin-dependent kinase cascade. The skeletal tissue stores Ca^2+^, and intracellular Ca^2+^ is a universal second messenger and sensed by Ca^2+^ receptor calmodulin [4]. Ca^2+^/CaM mediated signaling plays an important role in proliferation, differentiation and metabolism [5]. CaMKK2 is activated through Ca^2+^/CaM binding and in turn phosphorylates CaMKI and CaMKIV resulting in their full activation and forming a CaMK cascade [6]. Besides CAMKI and CAMKIV, adenosine monophosphate activated protein kinase (AMPK) is also a substrate of CaMKK2, which coordinates cellular energy balance [7].

CaMKK2 is expressed abundantly in brain, thus CaMKK2 has been studied intensively in relationship to the function and development of neurons. CaMKK2 over-activation induces dendritic spine loss, and inhibiting CaMKK2 activity protects hippocampal neurons [8]. CaMKK2 has a male-specific role in memory formation [9], and is involved in cerebellar granule cell development [10]. Besides neurons, the function of CaMKK2 in cell development has been investigated in other tissues. CaMKK2 stimulates homeostatic osteoclastogenesis [11] and inhibits adipogenesis [12]. In early myeloid progenitors, CaMKK2 functioned as an inhibitor of granulocytic fate commitment and differentiation [13]. Although the function of CaMKK2 in cell development has been investigated extensively in neurons, adipose and myeloid cells, the role of this kinase in the muscle is largely unknown. AMPK, as one of the substrates of CaMKK2, is able to mediate CaMKK2 action in various biological systems or cell types under either physiological or pathological conditions [8,14,15,16,17,18]. AMPK has been reported to be a regulator of myoblast differentiation. Satellite cell-specific AMPKα1 Knockout attenuated muscle regeneration and reduced myogenic differentiation in satellite cells. In contrast, Williamson.et al showed that AICAR-induced AMPK phosphorylation reduced the differentiation of myoblasts into myotubes through PGC1α transcription [19]. Based on these findings, we hypothesized that CaMKK2 might act as an upstream regulator of AMPK and play a role in regulating myogenesis and muscle regeneration.

To test our hypothesis, in the present study, we found that CaMKK2 is down-regulated in skeletal muscle in the cardiotoxin (CTX) or freeze-injury-induced muscle regeneration, indicating that CaMKK2 may play a role in skeletal muscle regeneration through regulating cell proliferation and differentiation. We also demonstrated that CaMKK2 is able to inhibit C2C12 cell proliferation and differentiation. Further study suggests that AMPK might mediate the action of CaMKK2 on myoblast proliferation and differentiation.

## 2. Results

### 2.1. CaMKK2 Expression Levels during Postnatal Myogenesis and Muscle Regeneration

**Figure 1:**
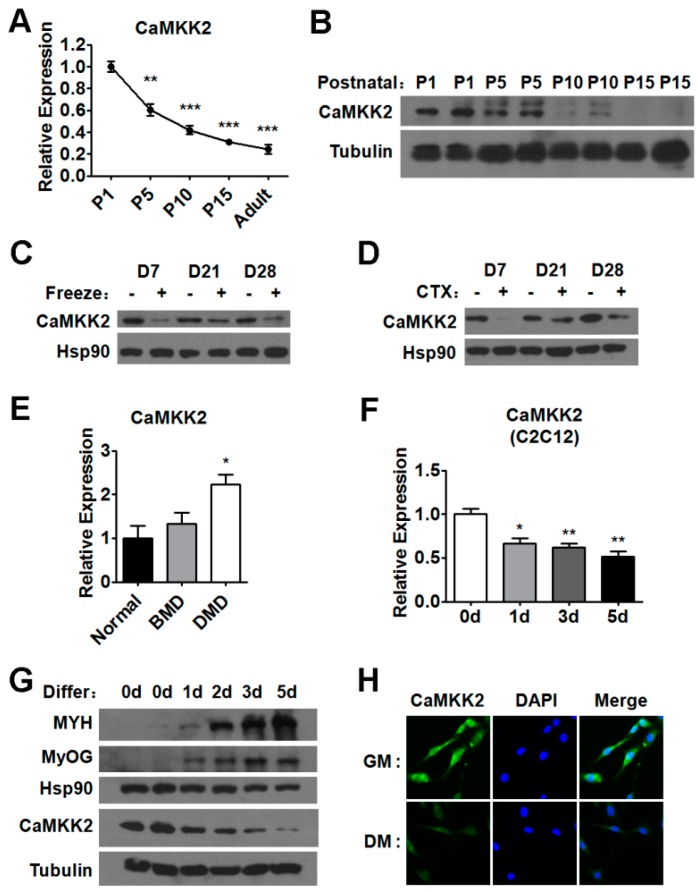
Expression levels of CaMKK2 in skeletal muscle during myogenesis and regeneration. (**A**) Quantitative RT-PCR analysis of CaMKK2 levels in postnatal myogenesis (*n* = 4); (**B**) Representative western blot showing the CaMKK2 protein levels in postnatal myogenesis; (**C**) Representative western blot showing the CaMKK2 protein in freeze injury-induced regeneration (*n* = 4); (**D**) Representative western blot showing the CaMKK2 protein levels in CTX injury-induced regeneration (*n* = 4); (**E**) Quantitative RT-PCR analysis of CaMKK2 levels in BMD or DMD patients (*n* = 3); (**F**) Quantitative RT-PCR analysis of CaMKK2 mRNA levels during C2C12 myoblasts differentiation (*n* = 3); (**G**) Western blotting analysis of CaMKK2, MYH and MyoG protein levels during C2C12 myoblasts differentiation; and (**H**) Immunofluorescence analysis of CaMKK2 was performed in C2C12 myoblasts and myotubes (GM, growth medium; DM, differentiation medium). Experiments were repeated at least twice. Means ± SEM (error bars) are shown. *, *p* < 0.05; **, *p* < 0.01; ***, *p* < 0.001.

Due to the low expression level of CaMKK2 in the skeletal muscle of adult mice, the role of CaMKK2 in muscle cells might have been underestimated (Figure S1A). We investigated the mRNA and protein levels of CaMKK2 in postnatal myogenesis, and found it decreased gradually and significantly (Figure 1A,B), indicating that CaMKK2 might have functions in myogenesis. We also examined the CaMKK2 expression level in two muscle injury models (Figure S1B,C). First we determined the expression of CaMKK2 in gastrocnemius muscles at 7, 21, and 28 days following freeze injury, and found that the protein levels of CaMKK2 were decreased after muscle damage (Figure 1C). Consistent with the results in freeze-injury model, CaMKK2 protein levels were decreased after CTX injury (Figure 1D). Meanwhile, we found that the mRNA levels of CaMKK2 were also down-regulated after injury in these two models (Figure S1D,E). These results suggested that CaMKK2 might be involved in the regulation of muscle regeneration. Duchenne muscular dystrophy (DMD) is the most prevalent neuromuscular disorders, which is characterized by progressive muscle weakness and wasting due to the absence of dystrophin protein that causes degeneration of skeletal muscle. We found that CaMKK2 mRNA level increased significantly in patients with DMD but not in patients with Becker muscular dystrophy (BMD) [20], which is the milder form of dystrophinopathy (Figure 1E). These results suggested that the increase in CaMKK2 levels might contribute to the impaired muscle regeneration and play a role in muscle degeneration in DMD.

To complement these in vivo expression studies, we examined CaMKK2 level during C2C12 myoblast differentiation, which is a widely used in vitro myogenesis model. We found that upon induction of differentiation both mRNA and protein levels of CaMKK2 were down-regulated (Figure 1F–H), while the expression levels of myogenic markers were increased as expected (Figure 1G and Figure S1F,G). These in vivo (muscle regeneration) and in vitro (myoblast differentiation) studies suggested that CaMKK2 is dynamically expressed during muscle differentiation and might play physiological and pathological roles during muscle regeneration.

### 2.2. CaMKK2 Inhibits C2C12 Myoblasts Proliferation

**Figure 2:**
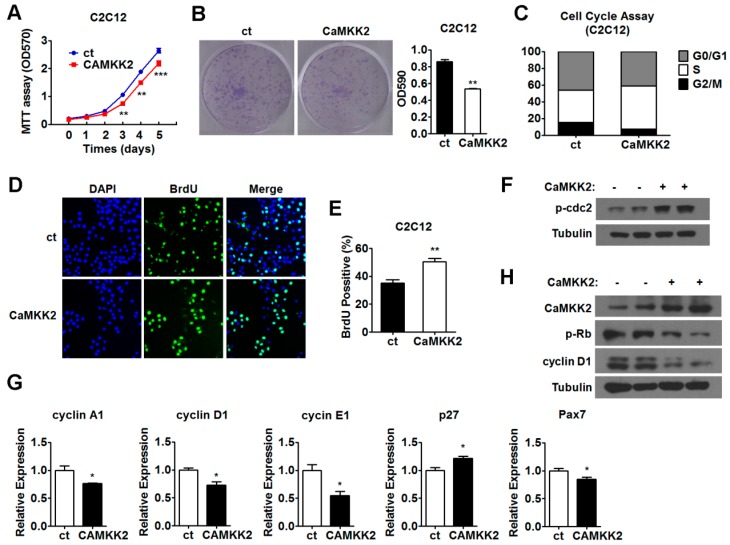
CaMKK2 inhibits C2C12 myoblasts proliferation. (**A**) MTT assay of the proliferation of C2C12 myoblasts transfected with CaMKK2 or control plasmids and maintained over a period of 5 days; (**B**) Representative micrographs of crystal violet-stained C2C12 cell colonies, which were transfected with CaMKK2 plasmids as indicated (**left**), absorbance of each well at 590 nm (**right**) (*n* = 3); (**C**) Flow cytometric analysis of the C2C12 myoblasts transfected with CaMKK2 plasmids during proliferation. Percentage of cells in G1, S and G2 phases were analyzed (*n* = 3); (**D**,**E**) DNA synthesis in C2C12 myoblasts transfected with CaMKK2 or control plasmids by BrdU incorporation assay. Representative images of BrdU immunofluorescence (**green**) and DAPI-stained nuclei (**blue**) (**D**); Quantification of the BrdU positive cells (**E**, *n* = 3); (**F**) Representative western blot showing the p-cdc2 Tyr15 protein levels in C2C12 myoblasts transfected with CaMKK2 plasmids; (**G**) Quantitative RT-PCR analysis of cyclin A1, cyclin D1, cyclin E1, p27 and Pax7 mRNA levels in C2C12 myoblasts transfected with CaMKK2 plasmids (*n* = 3); and (**H**) Representative western blot showing the cyclin D1 and p-Rb (Ser807/811) protein levels in C2C12 myoblasts transfected with CaMKK2 plasmids. Experiments were repeated at least twice. Means ± SEM (error bars) are shown. *, *p* < 0.05; **, *p* < 0.01, ***, *p* < 0.001.

Since myogenic proliferation and differentiation are two important processes during muscle regeneration, to test our hypothesis, we first investigated the effect of CaMKK2 on proliferation in C2C12 myoblasts. We found that transfection of a CaMKK2 expression plasmid was able to suppress C2C12 myoblasts proliferation as shown by MTT assay and crystal violet staining (Figure 2A,B). The transfection efficiency was evaluated by both Western blot and qRT-PCR analysis (Figure S2A,B). Cell cycle analysis by flow cytometry showed that, after CaMKK2 transfection, the proportion of G1 phase and G2 phase cells decreased, and the proportion of S phase cells increased compared with control group treated with empty vectors (Figure 2C). Consistently, we found that there were more BrdU positive cells after CaMKK2 transfection (Figure 2D,E), which reflected high rate of DNA synthesis, indicating that most part of cells stayed in S phase. Thus, our preliminary speculation was that CaMKK2 overexpression induced C2C12 cell S phase arrest and inhibited C2C12 myoblast proliferation. In accordance with these results, we found that cdc2-Tyr15 phosphorylation was increased by CaMKK2 overexpression (Figure 2F). During S phase, the activity of cdc2-Tyr15 kinase is high [21]. Some studies showed that cdc2-Tyr15 phosphorylation acted as a central mechanism for S phase arrest, when cells arrested in S phase, cdc2-Tyr15 phosphorylation was up-regulated [22]. Thus, this result was consistent with cell cycle assay, and further supported the notion that high levels of CaMKK2 expression could lead to S phase arrest in C2C12 cell line.

Furthermore, we analyzed the mRNA levels of cyclinA1, cyclin D1, cyclin E1, p27 and Pax7 in C2C12 cell line overexpressing CaMKK2, and found that the expression of cyclin A1, cyclin D1, cyclin E1 and Pax7 decreased, while the expression of p27 increased (Figure 2G). It is known that cyclin D1 is a protein required for progression through the G1 phase of the cell cycle [23], and the retinoblastoma protein (Rb), which is a tumor suppressor, is inactivated while Rb is phosphorylated to p-Rb (Ser807/811) [24]. We found that the protein levels of cyclin D1 and p-Rb (Ser807/811) decreased upon CaMKK2 overexpression (Figure 2H), suggesting that CaMKK2 overexpression could lead to cell cycle retardation. We also checked the effect of CaMKK2 overexpression on apoptosis in C2C12 cells but did not find any changes in Bcl-xL and cleaved caspase3 levels, suggesting that CaMKK2 overexpression affected cell proliferation not through reducing cell viability via inducing apoptosis (Figure S2C). Together, these results illustrated that CaMKK2 acted as a negative regulatory factor during C2C12 myoblast proliferation.

### 2.3. CaMKK2 Inhibits C2C12 Myoblasts Differentiation

**Figure 3:**
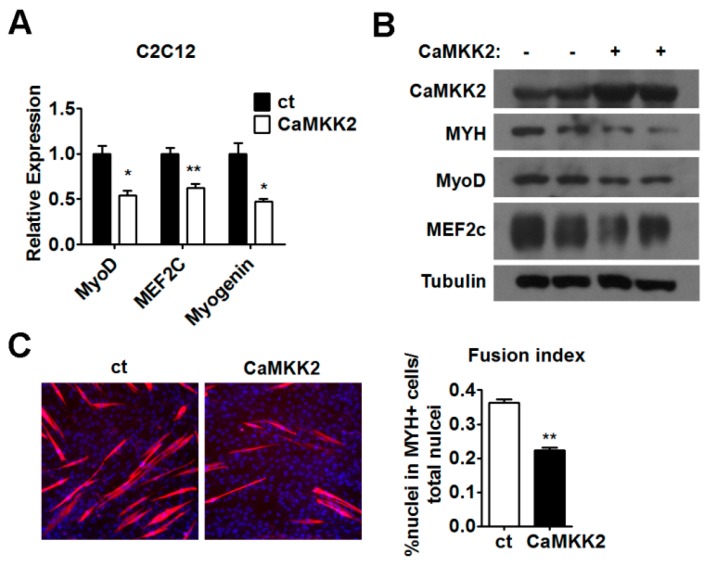
CaMKK2 inhibits C2C12 myoblasts differentiation. (**A**) Quantitative RT-PCR analysis of MyoD, MEF2c and Myogenin mRNA levels in C2C12 myotubes transfected with CaMKK2 plasmids (*n* = 3); (**B**) Representative western blot showing the MYH, MyoD and MEF2c protein levels in C2C12 myotubes transfected with CaMKK2 plasmids; and (**C**) Immunofluorescence analysis of MYH was performed in C2C12 myotubes 48 h after CaMKK2 plasmids transfection (**left**), quantification of the fusion index of indicated cells (**right**) (*n* = 3). Experiments were repeated at least twice. Means ± SEM (error bars) are shown. *, *p* < 0.05; **, *p* < 0.01.

Since the CaMKK2 expression was decreased in postnatal myogenesis (Figure 1A,B) and the expression of CaMKK2 mRNA and protein was negatively correlated with the levels of myogenic markers during myogenic differentiation (Figure 1F,G and Figure S1F,G), we speculated that CaMKK2 might have an effect on C2C12 cell differentiation. To test this hypothesis, C2C12 cells were transfected with CaMKK2 plasmids on the first day of differentiation. We found that mRNA levels of MyoD, MEF2c and Myogenin decreased by approximately 50% compared to the negative control group (Figure 3A), and the protein levels of MYH, MyoD and MEF2c decreased after CaMKK2 overexpression (Figure 3B). The MYH expression was also analyzed by immunofluorescence to examine the myotube formation in cultured C2C12 cells after 48 h transfection. We observed a decrease of myotube formation after CaMKK2 overexpression (Figure 3C). These results clearly demonstrated that CaMKK2 overexpression inhibited myoblast differentiation in vitro.

### 2.4. Inhibition of CaMKK2 Promotes Cell Proliferation and Differentiation

**Figure 4:**
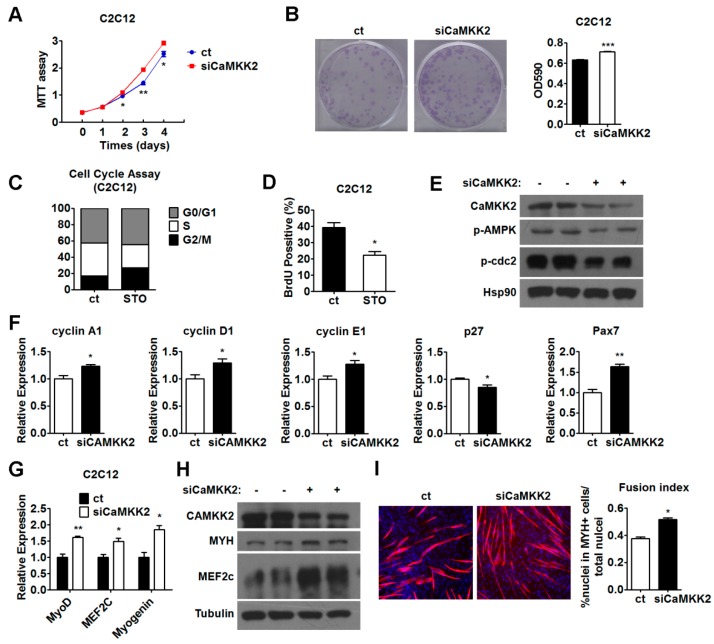
Down-regulation of CaMKK2 promotes C2C12 myoblasts proliferation and differentiation. (**A**) MTT assay of the proliferation of C2C12 myoblasts transfected with siCaMKK2 or control and maintained over a period of 5 days; (**B**) Representative micrographs of crystal violet-stained C2C12 cell colonies, which were transfected with siCaMKK2 as indicated (**left**), absorbance of each well at 590 nm (**right**) (*n* = 3); (**C**) Flow cytometric analysis of the C2C12 myoblasts treated with 10 μM STO-609 during proliferation. Percentage of cells in G1, S and G2 phases were analyzed (*n* = 3); (**D**) DNA synthesis in C2C12 cell treated with 10 μM STO-609 by BrdU incorporation assay. Quantification of the BrdU positive cells (*n* = 3); (**E**) Representative western blot showing the p-cdc2 Tyr15 protein level in C2C12 cells transfected with siCaMKK2; (**F**) Quantitative RT-PCR analysis of cyclin A1, cyclin D1, cyclin E1, p27 and Pax7 mRNA levels in the C2C12 myoblasts transfected with siCaMKK2 (*n* = 3); (**G**) Quantitative RT-PCR analysis of MyoD, MEF2c and Myogenin mRNA levels in the C2C12 myotubes transfected with siCaMKK2 (*n* = 3); (**H**) Representative western blot showing the MYH, MEF2c protein expression in C2C12 myotubes transfected with siCaMKK2; and (**I**) Immunofluorescence analysis of MYH was performed in C2C12 myotubes 48 h after siCaMKK2 transfection (**left**), quantification of the fusion index of indicated cells (**right**) (*n* = 3). Experiments were repeated at least twice. Means ± SEM (error bars) are shown. *, *p* < 0.05; **, *p* < 0.01, ***, *p* < 0.001.

To confirm the finding that CaMKK2 overexpression is able to suppress C2C12 myoblasts proliferation and differentiation, a knockdown approach using a CaMKK2-specific siRNA was employed. We observed a reduction of CaMKK2 mRNA and protein levels after transfection of a CaMKK2-specific siRNA (siCaMKK2) (Figure S2D,E). As expected, we observed that CaMKK2 knockdown promoted C2C12 myoblasts proliferation as shown by MTT analysis and crystal violet staining (Figure 4A,B). In addition, treatment of STO-609 [25], a specific inhibitor of CaMKK2 resulted in a decrease of the proportion of S phase cells compared with the control group (Figure 4C). Consistently, we found that there were less BrdU positive cells after STO-609 treatment (Figure 4D). In accordance with these results, we found that cdc2-Tyr15 phosphorylation was decreased after siCaMKK2 treatment (Figure 4E). Furthermore, we analyzed the mRNA levels of cell cycle-related genes and found that cyclin A1, cyclin D1, cyclin E1 and Pax7 were increased, while p27 was decreased (Figure 4F). Similar results were obtained when another CaMKK2-specific siRNA (siCaMKK2-b) was employed (Figure S2F,G). These data indicated that CaMKK2 inhibition could promote C2C12 myoblast proliferation.

The effect of CaMKK2-specific siRNA on the C2C12 myogenesis was also examined. We observed that the mRNA levels of MyoD, MEF2c and Myogenin increased by approximately 1.5-fold, and the protein levels of MYH and MEF2c increased in siCaMKK2-transfected C2C12 myotubes compared to the control groups (Figure 4G,H). The MYH expression was also analyzed by immunofluorescence to examine the myotube formation. We observed an increase of myotube formation compared to the control group (Figure 4I). Similar results were obtained when siCaMKK2-b was used (Figure S2H,I). These results clearly demonstrated that CaMKK2 knockdown could promote C2C12 myoblast differentiation. Together, we hypothesized that CaMKK2 negatively regulates myoblast proliferation and differentiation.

### 2.5. CaMKK2 Inhibits C2C12 Myoblasts Proliferation and Differentiation through AMPK

**Figure 5:**
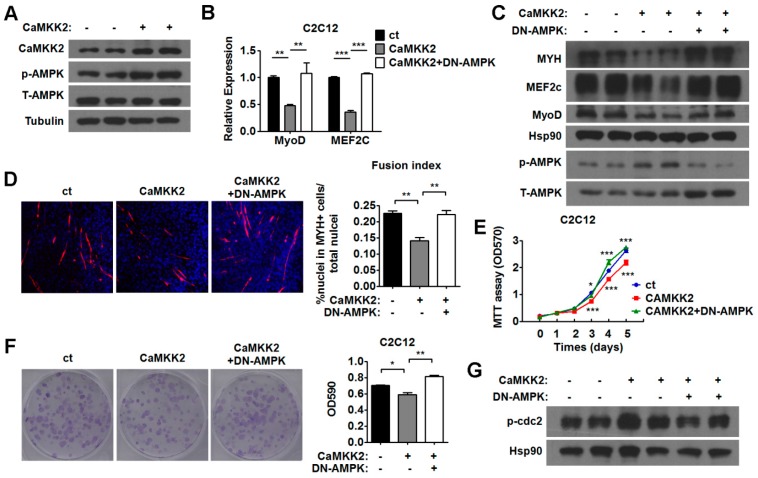
CaMKK2 inhibits C2C12 myoblasts proliferation and differentiation through AMPK activation. (**A**) Representative western blot showing the p-AMPK protein levels in the C2C12 cells transfected with CaMKK2 plasmids; (**B**) Quantitative RT-PCR analysis of MyoD, and MEF2c mRNA levels in C2C12 myotubes transfected with CaMKK2 plasmids and then treated with DN-AMPK adenovirus for 48 h (*n* = 3); (**C**) Representative western blot showing the MYH, MyoD and MEF2c protein levels in C2C12 myotubes transfected with CaMKK2 plasmids and then treated with DN-AMPK adenovirus for 48 h; (**D**) Immunofluorescence analysis of MYH was performed in C2C12 myotubes transfected with CaMKK2 plasmids and then treated with DN-AMPK adenovirus for 48 h (**left**), quantification of the fusion index of indicated cells (**right**) (*n* = 3); (**E**) MTT assay of the proliferation ability of C2C12 myoblasts transfected with CaMKK2 or control plasmids, and then treated with DN-AMPK adenovirus and maintained over a period of 5 days; (**F**) Representative micrographs of crystal violet-stained C2C12 cell colonies, which were transfected with CaMKK2 or control plasmids, and then treated with DN-AMPK adenovirus (**left**), absorbance of each well at 590 nm (**right**) (*n* = 3); and (**G**) Representative western blot showing the p-cdc2 Tyr15 protein levels in C2C12 myotubes transfected with CaMKK2 plasmids and then treated with DN-AMPK adenovirus for 48 h. Experiments were repeated at least twice. Means ± SEM (error bars) are shown. *, *p* < 0.05; **, *p* < 0.01; ***, *p* < 0.001.

CaMKK2 has been reported as an AMPK kinase [26,27,28]. We noticed that the levels of p-AMPK was decreased in siCaMKK2-transfected C2C12 myotubes (Figure 4E) and the reduced CaMKK2 protein expression in differentiated C2C12 cells was accompanied by a reduction in p-AMPK levels (Figure S2J). Based on these observations, we speculated that the phosphorylation of AMPK could be regulated by CaMKK2 in muscle cells and AMPK might mediate the action of CaMKK2 in muscle cells. To test this hypothesis, we first investigated the effect of AMPK activation on myoblast differentiation and proliferation. We found that activation of AMPK by the treatment of agonist AICAR [29] and A-769662 [30,31] could decrease MYH protein levels (Figure S3A,B), while inhibition of AMPK by either DN-AMPK (a dominant negative AMPK) or Compound C (an AMPK inhibitor) could elevate the protein levels of MYH in C2C12 myotubes (Figure S3C,D). These results were in agreement with recent studies showing that AMPK inhibits myoblast differentiation [19]. In addition, some studies showed that AICAR could lead to S phase arrest in retinoblastoma cells [32]. In this study, we found that CaMKK2 also could inhibit myogenesis and promote S phase arrest in C2C12 myoblasts (Figure 2C). Based on these findings, we speculated that CaMKK2 might regulate myoblast proliferation and differentiation through activating AMPK. To test whether AMPK mediates the CaMKK2 effect on C2C12 proliferation and differentiation, we first checked the phosphorylation status of AMPK after CaMKK2 overexpression. As expected, p-AMPK protein levels increased in C2C12 myoblasts after overexpressing CaMKK2 (Figure 5A), suggesting that CaMKK2 could activate AMPK in C2C12 cells, and CaMKK2 overexpression might suppress C2C12 myoblast proliferation and differentiation through AMPK activation.

To validate whether AMPK mediates the action of CaMKK2 during myoblast proliferation and differentiation, we inhibited the AMPK activation by DN-AMPK in C2C12 myotubes transfected with CaMKK2. We found that the mRNA expression of MyoD and MEF2c, and the protein expression of MYH, MyoD and MEF2c were derepressed by DN-AMPK treatment in CaMKK2-transfected C2C12 myotubes (Figure 5B,C). We also observed that DN-AMPK treatment restored the myotube formation in CaMKK2-transfected C2C12 cells (Figure 5D). These results suggested that inhibition of AMPK by DN-AMPK was able to attenuate the inhibitory effect of CaMKK2 on C2C12 myogenesis. Similarly, MTT assay and crystal violet staining results showed that inhibition of AMPK by DN-AMPK could abolish the suppressive effect of CaMKK2 on C2C12 myoblast proliferation (Figure 5E,F). As expected, we found that DN-AMPK treatment abrogated the effect of CaMKK2 overexpression on cdc2-Tyr15 phosphorylation (Figure 5G). Similar results were obtained when Compound C was used to inhibit AMPK in C2C12 myotubes overexpressing CaMKK2 (Figure S4A–F). These results indicated that AMPK might mediate the action of CaMKK2 during myoblast proliferation and differentiation.

We also tested whether activation of AMPK by AICAR or A-769662 is able to antagonize the effect of CaMKK2 knockdown on myoblast differentiation and proliferation. After we verified the inhibition of AMPK by siCaMKK2 transfection in C2C12 cells (Figure 4E), we found that either AICAR or A-769662 treatment could decrease the mRNA levels of myogenic markers and suppress myotube formation in C2C12 cells transfected with siCaMKK2 (Figure S5A–E). Similarly, we found that both AICAR and A-769662 administration could decrease proliferation in C2C12 myoblasts transfected with siCaMKK2 (Figure S5F–I). Together, these results further supported the notion that AMPK mediates the effect of CaMKK2 on myoblast proliferation and differentiation.

### 2.6. AMPK Pathways Mediate the Effect of CaMKK2 on Myoblasts Proliferation and Differentiation

**Figure 6:**
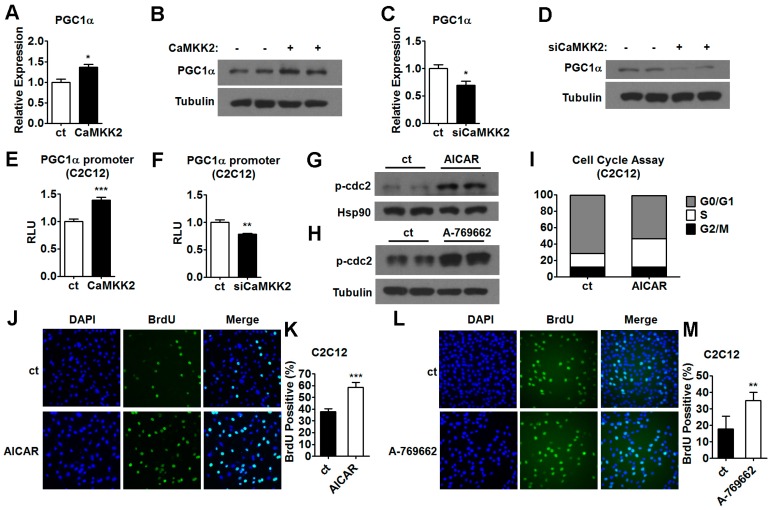
The mechanism of AMPK inhibits C2C12 myoblasts proliferation and differentiation. (**A**) Quantitative RT-PCR analysis of PGC1α mRNA levels in C2C12 myotubes transfected with CaMKK2 plasmids (*n* = 3); (**B**) Representative western blot showing the PGC1α protein levels in the C2C12 cells transfected with CaMKK2 plasmids; (**C**) Quantitative RT-PCR analysis of PGC1α mRNA levels in C2C12 myotubes transfected with siCaMKK2 (*n* = 3); (**D**) Representative western blot showing the PGC1α protein levels in the C2C12 cells transfected with siCaMKK2; (**E**) PGC1α transcriptional activity in C2C12 cells co-transfected with CaMKK2 and PGC1α promoter plasmids (*n* = 3); (**F**) PGC1α transcriptional activity in C2C12 cells co-transfected with siCaMKK2 and PGC1α promoter plasmids (*n* = 3); (**G**,**H**) Representative western blot showing the p-cdc2 Tyr15 protein levels in C2C12 myoblasts treated with 0.25 mM AICAR (**A**,**G**,**I**) and 80 μM A-769662 (**H**) for 48 h; (**I**) Flow cytometric analysis of C2C12 myoblasts treated with 0.25 mM AICAR for 48 h during proliferation. Percentage of cells in G1, S and G2 phases were analyzed (*n* = 3); (**J**,**L**) DNA synthesis in C2C12 cells treated with 0.25 mM AICAR (J) or 80 μM A-769662 (**L**) by BrdU incorporation assay; (**K**,**M**) Quantification of the BrdU positive cells (*n* = 3) in C2C12 cells treated with 0.25 mM AICAR (**K**) or 80 μM A-769662 (**M**) by BrdU incorporation assay. Experiments were repeated at least twice. Means ± SEM (error bars) are shown. *, *p* < 0.05; **, *p* < 0.01; ***, *p* < 0.001.

The molecular mechanism underlying the effect of AMPK on muscle cells proliferation and differentiation remains unclear. It has been proposed that AMPK inhibits myoblast differentiation through a PGC1α-dependent mechanism, and AMPK regulates PGC1α gene expression [19,33]. We found that the expression levels of PGC1α increased after CaMKK2 overexpression in C2C12 cells (Figure 6A,B). In contrast, downregulation of CaMKK2 by specific siRNA decreased the expression levels of PGC1α in C2C12 cells (Figure 6C,D). In addition, an increase in the mRNA levels of PGC1α was observed in the gastrocnemius muscle of mice (Figure S6A,B). We also observed that AICAR treatment led to an increase of PGC1α mRNA level in C2C12 cells (Figure S6C). To determine whether CaMKK2 activates PGC1α transcription, we generated a 2 kb PGC1α promoter. Using Luciferase reporter assay, we found that CaMKK2 overexpression increased the promoter activity of PGC1α, while CaMKK2 knockdown decreased it (Figure 6E,F). These results suggested that CaMKK2 could positively regulate PGC1α expression, and CaMKK2 might inhibit C2C12 cell differentiation through AMPK-PGC1α pathway.

The mechanism of AMPK in proliferation is not known in C2C12 muscle cells. We found that both AICAR and A-769662 could promote cdc2-Tyr15 phosphorylation in C2C12 myoblasts (Figure 6G,H). In agreement with these findings cell cycle analysis using flow cytometry revealed that the proportion of S phase cells increased in these ACIAR-treated C2C12 cells (Figure 6I). Consistently, we found that the number of BrdU positive cells increased after either AICAR or A-769662 administration as compared to control groups (Figure 6J–M). Based on these findings, we proposed that CaMKK2 might inhibit C2C12 cell proliferation through AMPK-cdc2 pathway.

It has been shown that CaMKK2 can form a complex with and activate AMPK, while a K193A mutation in CaMKK2 disrupts the binding to AMPK, but not CaMKIV [34]. We found that this CaMKK2 K193A mutant (CaMKK2 mut) could not activate AMPK in C2C12 cells (Figure S7A). More interestingly, we found that overexpression of CaMKK2 mut could neither lead to cell cycle retardation nor inhibit myoblast differentiation C2C12 cells (Figure S7B–D). These results indicated that the binding of CaMKK2 to AMPK and the activation of AMPK might be required for the regulation of proliferation and differentiation by CaMKK2 in C2C12 muscle cells.

### 2.7. Overexpression of CaMKK2 Inhibits Muscle Regeneration in Vivo

**Figure 7:**
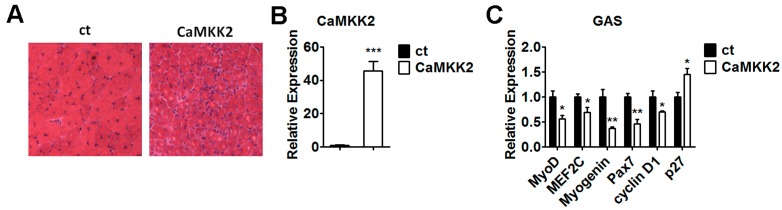
CaMKK2 inhibits muscle regeneration in mice. (**A**) H&E staining of regenerating gastrocnemius muscles from mice after electroporation with CaMKK2 and control plasmids at 14 days following freeze injury; (**B**) Quantitative RT-PCR analysis of CaMKK2 mRNA levels in gastrocnemius muscles (GAS) after electroporation with CaMKK2 and control plasmids at 14 days following freeze injury; and (**C**) Quantitative RT-PCR analysis of MyoD, MEF2c, Myogenin, Pax7, cyclin D1 and p27 mRNA levels in gastrocnemius muscles (GAS) after electroporation with CaMKK2 and control plasmids at 14 days following freeze injury (*n* = 3). Means ± SEM (error bars) are shown. *, *p* < 0.05; **, *p* < 0.01; ***, *p* < 0.001.

The in vitro findings that CaMKK2 is able to affect C2C12 cell proliferation and differentiation through AMPK activation promoted us to check the effect of CaMKK2 on skeletal muscle regeneration. Gastrocnemius muscle was transfected with CaMKK2 plasmids by electroporation. After freeze injury, muscle histology was examined at day 14 after electroporation (Figure 7A). The CaMKK2 transfection efficiency was evaluated by qRT-PCR analysis (Figure 7B). We found that muscle regeneration in CaMKK2 overexpressing gastrocnemius muscles was dramatically impaired compared to the control muscles. Consistently, we found that the mRNA levels of differentiation and proliferation markers, including MyoD, MEF2c, Myogenin, Pax7 and cyclin D1were decreased significantly, while p27 was increased compared to the control group (Figure 7C). These in vivo results indicated that overexpression of CaMKK2 inhibited skeletal muscle regeneration.

## 3. Discussion

Skeletal muscle has a major role in locomotion, and functions as metabolism regulatory entities. Skeletal muscle regeneration in adults occurs upon muscle injury. When injury happens, the satellite cells will be activated, and proliferate to muscle progenitor cells and differentiate to form myofibers. Most of muscle disorders are associated with impaired regenerative potential of muscle tissues.

It has been reported that CaMKK2 plays an important role in neuron development [10], adipogenesis [12] and cancer progression [35,36,37,38,39,40], however, its function in skeletal muscle differentiation and regeneration remains unknown. In this study, we employed a specific CaMKK2 antibody (Sangon Biotech. NO. D153544, Shanghai, China), which was confirmed by western blot analysis (Figure S8A), to evaluate the protein levels of CaMKK2 during muscle regeneration. We found that the protein and mRNA levels of CaMKK2 were down-regulated in freeze or CTX-induced injury in the skeletal muscle of mice (Figure 1C,D and Figure S1D,E). We also found the CaMKK2 mRNA levels increased in DMD patients (Figure 1E). These results indicated that CaMKK2 might play a regulatory role in muscle regeneration and abnormal expression of CaMKK2 might lead to impaired muscle regeneration. Since myoblast proliferation and differentiation are important for muscle regeneration, we hypothesized that CaMKK2 might play an important role in the myoblast proliferation or differentiation during muscle regeneration. To test this hypothesis, CaMKK2 was overexpressed or knocked down by transfected with CaMKK2 plasmid or siRNA respectively. When CaMKK2 was overexpressed on the first day of myogenic differentiation, the expression levels of myogenic markers (MyoD, MEF2c, Myogenin, and/or MYH) significantly decreased in C2C12 cells (Figure 3A–C). These data showed that CaMKK2 inhibited myogenic differentiation. Consistently, CaMKK2 knockdown increased the expression of these myogenic markers (Figure 4G–I). Meanwhile, we observed an inhibition of C2C12 myoblast proliferation by CaMKK2 overexpression and a promotion of C2C12 myoblast proliferation by CaMKK2 inhibition (Figure 2A–D and Figure 4A–D). To be noted, since the proliferative inhibition effect of CaMKK2 was not strong, the effect of CaMKK2 on myoblast differentiation might play a crucial part in muscle regeneration.

CaMKK2 has three substrates, CaMKI, CaMKIV and AMPK. Since it has been shown that the expression levels of CaMKI and CaMKIV are very low in skeletal muscle [41,42], we first tested whether AMPK could mediate the action of CaMKK2 and found that AMPK was able to mediate the effect of CaMKK2 on C2C12 cell proliferation and differentiation. Interestingly, we found that a CaMKK2 mut, which could not interact with and activate AMPK [34], was not able to lead to cell cycle retardation or inhibit myoblast differentiation as the wild type CaMKK2 did (Figure S7A–D). Since this K193A mutation only disrupts the binding to AMPK, but not CaMKIV, we speculated that CaMKIV might not be involved in the regulation of myoblast proliferation and differentiation. However, further studies are required to test whether and/or how CaMKI and CaMKIV mediate the action of CaMKK2 in muscle cell proliferation and differentiation.

AMPK is one of the substrates of CaMKK2, and plays a role in cellular energy homeostasis. Recent studies show that AMPK inhibits myoblast differentiation [19,33]. Therefore, we speculated that CaMKK2 might inhibit C2C12 cell proliferation and differentiation through AMPK activation. To test this possibility, we used AMPK agonist AICAR or A-769662 and AMPK inhibitor Compound C or DN-AMPK to manipulate the activity of AMPK. As we expected, DN-AMPK and Compound C could abolish the effect of CaMKK2 during differentiation or proliferation (Figure 5A–F and Figure S4A–F), while AICAR or A-769662 could abrogate the effect of CaMKK2 knockdown in C2C12 cells (Figure S5A–C,F,G).

The role of AMPK in muscle differentiation or regeneration and the underlying mechanisms are not very clear. Fu, X. et al. [43,44] showed that AMPKα1 activity was necessary to promote Myogenin expression and myogenesis, and satellite cell-specific AMPKα1 Knockout attenuated muscle regeneration and reduced myogenic differentiation in satellite cells. In contrast some studies show that AMPK inhibits myoblast differentiation though PGC1α transcription, and demonstrates that AICAR-induced AMPK phosphorylation reduces differentiation of myoblasts into myotubes through PGC1α transcription [19]. Regeneration in CTX-treated tibialis anterior muscle was dramatically impaired in mice treated with AICAR [45]. Here, we showed that AMPK activation by CaMKK2 (Figure 5A) in C2C12 cells was accompanied by an elevation of PGC1α expression (Figure 6A,B) and a suppression of myoblast differentiation (Figure 3A–C), while AMPK inactivation by the knockdown of CaMKK2 expression (Figure 4E) in C2C12 cells was accompanied by a reduce in PGC1α expression (Figure 6C,D) and an increase in myoblast differentiation (Figure 4G,H,I). These results suggested that the activation of AMPK by CaMKK2 could lead to an inhibition of myoblast differentiation and AMPK/PGC1α pathway might mediate the effect of CaMKK2 on myoblast differentiation.

The effect of AMPK on proliferation and the molecular mechanisms involved are also not clear in C2C12 myoblasts. We found that AMPK activation by AICAR treatment promoted cdc2-Tyr15 phosphorylation. It has been reported that during S phase, the activity of cdc2-Tyr15 kinase is held high, when DNA replication is completed, cdc2-Tyr15 will be dephosphorylated and activated [21]. It also has demonstrated that treatment of various cell lines with AICAR led to arrest either in the G1 phase or S phase, suggesting the effects of AICAR on cell cycle progression depend on the cell type [32]. When C2C12 myoblasts were treated with AICAR or A-769662, we observed an increase in the S phase, which was accompanied by the phosphorylation of cdc2-Tyr15 (Figure 6G,H). These results indicated that CaMKK2-AMPK pathway inhibited C2C12 cell proliferation through cdc2 hyper-phosphorylation.

Since CaMKK2 is not a tyrosine kinase, we speculated that the effect of CaMKK2 overexpression on the tyrosine phosphorylation of cdc2 should be mediated through an indirect mechanism. It has been reported that Cdc25c is a phosphatase, which could dephosphorylate cdc2 [46]. In addition, AICAR could down-regulate Cdc25c levels, which led to cdc2 phosphorylation [46]. Other studies showed that some members of calcium/calmodulin-dependent protein kinase family could block cell division through inactivation of Cdc25 by phosphorylation, which might lead to a defect in cdc2 dephosphorylation [47]. These findings suggest that the overexpression of CaMKK2 might lead to an increase of cdc2 phosphorylation though CaMKK2 downstream signaling such as AMPK or CaMKI indirectly. Further studies are required to understand the molecular mechanism.Macrophages are key players in muscle regeneration, since they are able to control myogenesis and extracellular matrix remodeling [48]. In DMD, macrophage infiltration perpetuates and leads to progressive fibrosis [49]. We evaluated the presence of macrophages by CD68 staining in muscle samples from DMD patients. As expected, macrophage infiltration could be observed (Figure S8B). It is known that macrophages express CaMKK2 and the amplitude of macrophage inflammatory response is regulated by CaMKK2 [50]. However, whether the macrophage infiltration and the expression of CaMKK2 in the macrophage also contributed to the increase of CaMKK2 expression that observed in the muscle biopsies of DMD patients is unknown. We compared the protein levels of CaMKK2 in C2C12 cells and RAW264.7 macrophages with or without LPS treatment and found that the protein expression of CaMKK2 was slight higher in RAW264.7 macrophages than that in C2C12 cells (Figure S8C). These data suggested that the macrophage infiltration might only partially contribute to the elevation of CaMKK2 expression in the muscle biopsies of DMD patients. Given that the number and size of macrophages are relatively small as compared with the number and size of myotubes, we speculated that the elevation of CaMKK2 expression observed in the muscle biopsies form DMD patients might be mainly attributed to an increase in CaMKK2 expression in muscle cells. Further studies are required and special approaches such as laser capture microdissection and CaMKK2 tissue specific knockout mice may help to address this issue.

The macrophage accumulation was also observed in the freeze-injured muscle (Figure S8D), which is in agreement of the notion that resident macrophages in skeletal muscle become activated when muscle fibres are damaged [51]. The presence of macrophages in the muscle tissues overexpressing CaMKK2 on day 14 following freeze injury was also examined. As shown in Figure S8E, more macrophage accumulation was observed in the muscle of mice transfected with a CaMKK2 construct than that in control group which was transfected with a control plasmid. This result also indicated that the muscle regeneration could be inhibited by CaMKK2 overexpression and the abnormal up-regulation of CaMKK2 in the muscle of DMD patients might have harmful impact on the muscle regeneration.

Taken together, the mechanism of CaMKK2-AMPK pathway in C2C12 cells discovered in this study is important to enhance our understanding of muscle regeneration. As CaMKK2 levels are up-regulated in DMD patients, CaMKK2 inhibition maybe serve as a potential target to maintain skeletal muscle functions and provide a therapeutic strategy to treat patients with muscle injury or severe myopathies.

## 4. Methods

### 4.1. Cell Culture and Differentiation

Mouse C2C12 cell lines and HEK 293T were purchased from Cell Bank of Shanghai Institute of Cell Biology, Chinese Academy of Sciences, and cultured in DMEM with 10% fetal bovine serum. C2C12 myoblasts were induced to differentiate using low serum DMEM supplemented with 2% horse serum when the C2C12 myoblasts reached 80% confluence. AICAR was purchased from Sigma (St. Louis, MO, USA), A-769662 was purchased from Selleck (Shanghai, China), AMPK Inhibitor, and Compound C was purchased from Merck (Darmstadt, Germany).

### 4.2. CaMKK2 Expression Plasmid Construction

RNA samples from mouse muscle were reverse-transcribed to cDNA, and full-length CaMKK2 cDNA was amplified and cloned into the pcDNA3.1 plus vector using the following primers: Forward primer: CCCAAGCTTATGTCATCATGTGTCTCTAGCC, Reverse primer: GCTCTAGATCACAAGAGCACTTCCTCCTC.

### 4.3. DNA and siRNA Transfection

C2C12 cells were seeded in six-well plates. CaMKK2 or CaMKK2 siRNA was transfected on the first day of myogenic differentiation. After 6 h of transfection, medium was replaced with fresh DMEM supplemented with 2% horse serum, and samples were collected for quantitative RT-PCR and western blotting 48 h later. Two different CaMKK2 siRNAs were purchased from Genepharma. The sequence of CaMKK2 siRNAs: (a) 5’-CAGGAGAUUGCUAUCCUCAAAtt-3’, (b) 5’-GGUCGAGAAUUCAGUCAAAtt-3’.

### 4.4. Luciferase Reporter Assay

Luciferase assays were performed using the Dual-Luciferase Reporter Assay System (Promega, Madison, WI, USA) according the manufacturer’s instructions. Samples were collected 48 h later. Luciferase activities were measured on a luminometer (Berthold Technologies, Bad Wildbad, Germany). PGC1α promoter was cloned into pGL3 basic using the following primers: Forward primer: CGGGGTACCTGTGAAAACTGCAGATTTGA, Reverse primer: CCGCTCGAGGGGATTAATTCAGCTTTTGA.

### 4.5. RNA Isolation and Real-Time PCR Analysis

TRIzol reagent was used to extract total RNA from cell cultures according to the manufacturer’s instructions. Complementary DNAs were synthesized using PrimeScript RT reagent Kit (TaKaRa, Tokyo, Japan), and real-time PCR were performed by ABI Real-Time System (Applied Biosystems, Foster, CA, USA). The real time PCR primers of genes are listed in Table S1.

### 4.6. Western Blotting

C2C12 cell protein extracts were isolated using RIPA buffer containing phosphatase and protease inhibitor. The lysates were separated by SDS-PAGE, and transferred onto PVDF membranes, and probed with the various antibodies. Antibodies to myogenin (MyOG), MyoD, MEF2c and myosin heavy chain (MYH) were purchased from Santa Cruz Biotechnology. Antibodies to p-cdc2 (Tyr15), p-Rb (ser807/811), Cyclin D1, p-AMPKα (Thr172), AMPKα and Hsp90 were purchased from Cell Signaling Technology. Antibody to CaMKK2 was purchased from Sangon Biotech. CD68 antibody used in immunofluorescence was purchased from Bio-Rad. Western blots were scanned and intensities were determined using ImageJ (US National Institutes of Health, Bethesda, MD, USA). The quantitation results were provided in Table S2.

### 4.7. Immunocytochemistry

The immunocytochemistry was performed as described before with modification [52]. C2C12 myoblasts cultured in 12-well plates were fixed in 4% paraformaldehyde for 10 min, followed by 0.1% Triton X-100 for 5 min. Cells were incubated with MYH antibody at 4 °C overnight. Next, secondary goat anti-mouse IgG (Alexa Fluor 596; Invitrogen) was added, and cells were incubated at 37 °C for 60 min. Nuclei were stained with DAPI for 10 min. Images were taken with a fluorescent microscope. Fusion index was calculated as the number of nuclei incorporated with MYH versus the total number of nuclei.

### 4.8. Cell Proliferation Assay

Cell proliferation was measured by MTT assay and BrdU incorporation assay.

MTT assay: Cells were seeded into 96-well plates and treated with plasmids or drugs, then incubated with 0.5 mg/mL MTT for 4 h at 37 °C. The absorbance was measured at 570 nm using a multiwell spectrophotometer (Molecular Devices, Inc., Toronto, ON, Canada). All experiments were performed at least three times.

BrdU incorporation assay: Cells were incubated with 10 mg/mL BrdU (Sigma, St. Louis, MO, USA) for 2 h, then fixed with cold methanol. The fixed cells were treated with 2 N HCl/1% Triton X-100 for 30 min and then incubated with anti-BrdU antibody (Santa Cruz Biotechnology, SantaCruz, CA, USA) overnight at 4 °C. The cells were incubated for 1 h at room temperature with Alexa Fluor 488 goat anti-mouse IgG1 (1:1000, Invitrogen, Carlsbad, CA, USA). Cell nuclei were counterstained with DAPI (Thermo Fisher, Waltham, MA, USA) for 5 min. Images were taken with a fluorescent microscope (Olympus America Inc., Melville, NY, USA).

### 4.9. Cell Cycle Analysis

Cell cycle analysis was performed 48 h after transfection. Cells were trypsinized, washed with PBS, then fixed with cold 70% ethanol, and stored at 4 °C overnight. The fixed cells were resuspended in PBS at the density of 1 × 10^6^ cells/mL and incubated with RNase A (100 mg/mL) and stained with propidium iodide (50 mg/mL, Sigma) for 60 min in the dark. Then cells were subsequently analyzed by flow cytometry.

### 4.10. Muscle Regeneration

CTX injury model was induced by intramuscular injection of cardiotoxin. Freeze injury model was induced by a single freeze injury of the gastrocnemius (GAS) muscles as described before subject to slightly modification [53].

### 4.11. Statistical Analysis

Results are expressed as mean ± SEM of at least three independent experiments. Statistical significance was assessed by Student’s *t*-test for two groups. *p* values of 0.05 or less were considered statistically significant. The software used is GraphPad Prism 5.0 (GraphPad, San Diego, CA, USA).

## 5. Conclusions

In summary, our studies demonstrated that CaMKK2, which is down-regulated after muscle injury and decreased quickly in postnatal myogenesis, plays an inhibitory role in skeletal muscle cell proliferation and differentiation. Using AMPK agonist and inhibitor, we confirmed that AMPK mediates the function of CaMKK on muscle cell proliferation and differentiation. Importantly, our in vivo data suggested that CaMKK2 is able to inhibit gastrocnemius muscles regeneration in mice.

## Supplementary Materials

Supplementary materials can be found at www.mdpi.com/1422-0067/17/10/1695/s1.

## Abbreviations

AICAR	5-Aminoimidazole-4-carboxamide1-β-d-ribofuranoside
AMPK	adenosine monophosphate activated protein kinase
BMD	Becker muscular dystrophy
BrdU	5-Bromo-2-deoxyUridine
CaMK	Ca2+/calmodulin-dependent protein kinase
CaMKK2	Calcium/calmodulin-dependent protein kinase kinase 2
cdc2	cell division cycle protein 2 homolog
cDNA	complementary deoxyribonucleic acid
CREB	cAMP response element-binding protein
CTX	cardiotoxin
DAPI	4’,6-diamidino-2-phenylindole
DMD	duchenne muscular dystrophy
DMEM	Dulbecco minimum essential medium
GAS	gastrocnemius; HDAC5: histone deacetylase 5
Hsp90	heat shock protein 90
MEF2C	myocyte-specific enhancer factor 2C
mTOR	mammalian target of rapamycin
MTT	3-(4,5-dimethylthiazol-2-yl)-2,5-diphenyltetrazolium bromide
MYH	myosin heavy chain
MyoD	myogenic differentiation protein
MyoG	myogenin; PBS: phosphate-buffered saline
PCR	polymerase chain reaction
PGC1α	peroxisome proliferator-activated receptor-γ coactivator-1α
Rb	retinoblastoma protein; S6K: ribosomal protein S6 kinase
