# Supplementary material for: CaMKK2 Suppresses Muscle Regeneration through the Inhibition of Myoblast Proliferation and Differentiation
Source: Int J Mol Sci. 2016 Oct 24;17(10):1695. doi: 10.3390/ijms17101695 (PMC5085727; doi:10.3390/ijms17101695)
Supplement: Supplementary file 1 [file ijms-17-01695-s001.pdf]

Supplementary Materials: CaMKK2 Suppresses Muscle Regeneration through the Inhibition of Myoblast Proliferation and Differentiation

Cheng Ye, Duo Zhang, Lei Zhao, Yan Li, Xiaohan Yao, Hui Wang, Shengjie Zhang, Wei Liu, Hongchao Cao, Shuxian Yu, Yucheng Wang, Jingjing Jiang, Hui Wang, Xihua Li and Hao Ying

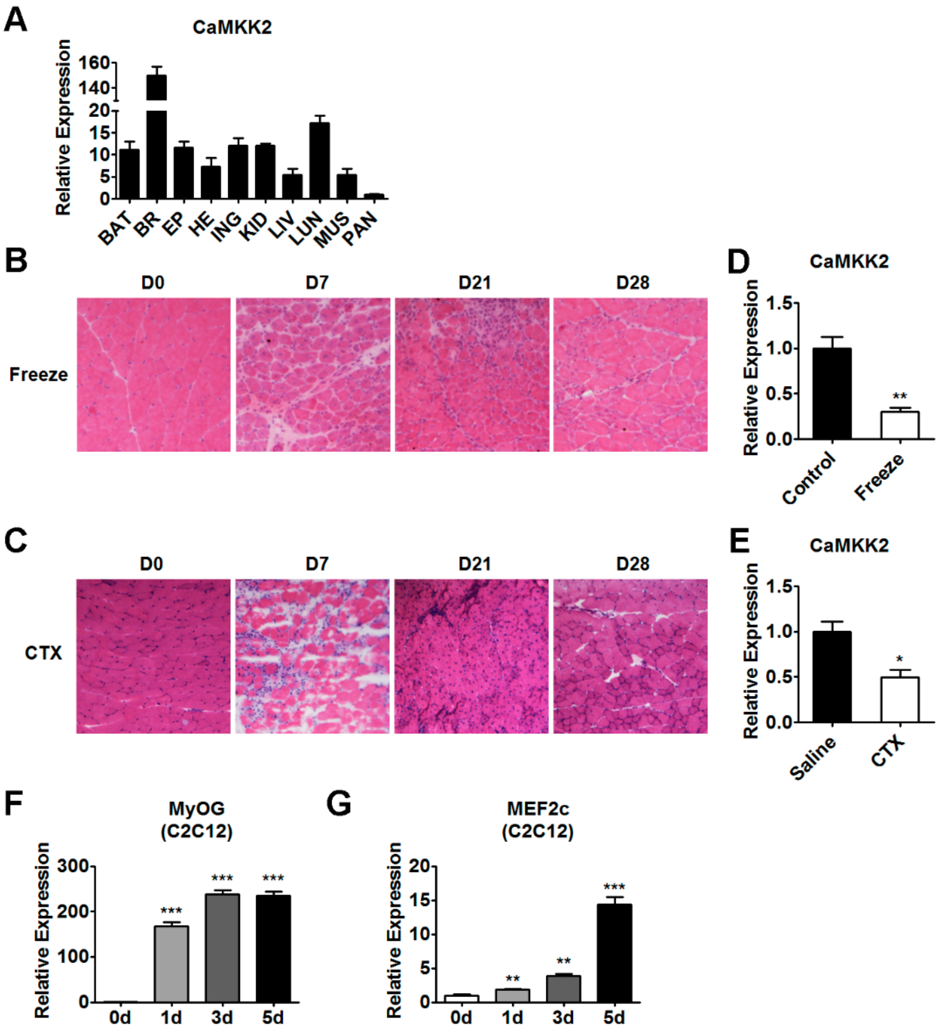

Figure S1. The expression of CaMKK2 in different tissues and myogenic markers during C2C12 myoblasts differentiation. (**A**) Quantitative RT-PCR analysis of CaMKK2 mRNA levels in different tissues as indicated (n = 3) (BAT: brown adipose tissue; BR: brain; EP: epididymal fat; HE: heart; ING: inguinal fat; KID: kidney; LIV: liver; LUN: lung; MUS: muscle; PAN: pancreas); (**B**,**C**) H&E staining of regenerating gastrocnemius muscles from mice at 7, 21 and 28 days following freeze injury (**B**) or CTX injury (**C**); (**D**,**E**) Quantitative RT-PCR analysis of CaMKK2 mRNA levels in freeze injury-induced regeneration (n = 4) or in CTX injury-induced regeneration (n = 4); (**F**,**G**) Quantitative RT-PCR analysis of myogenin (MyOG) (**F**) and MEF2c (**G**) mRNA levels during C2C12 myoblasts differentiation (n = 3). Means ± SEM (error bars) are shown. *, $p < 0.05$, **, $p < 0.01$; ***, $p < 0.001$.

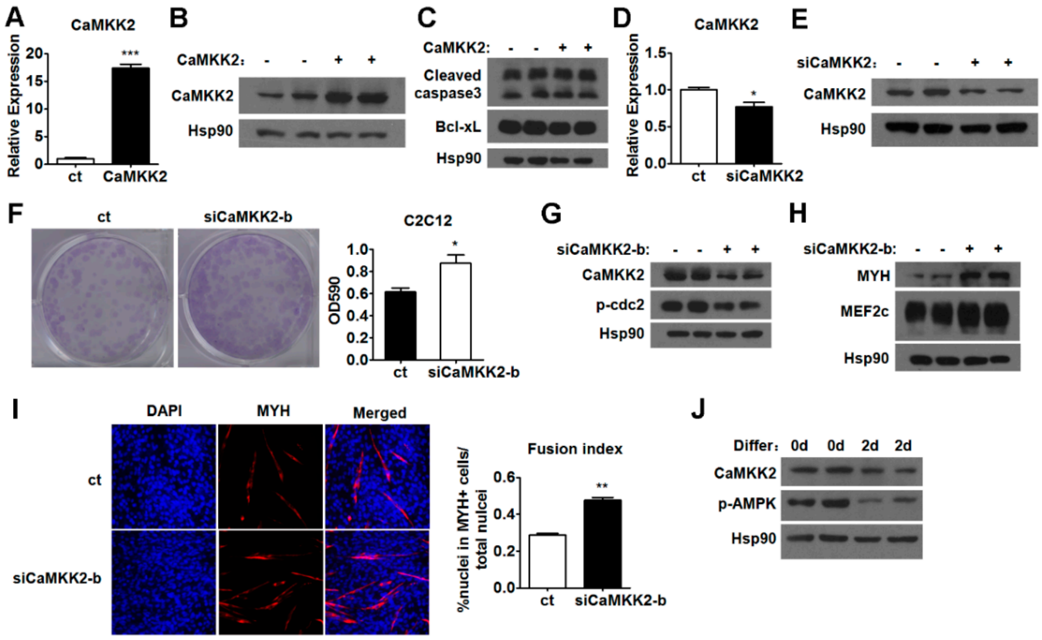

Figure S2. CaMKK2 levels in C2C12 myoblasts and p-AMPK protein levels in undifferentiated and differentiated C2C12 cells. (**A**) CaMKK2 mRNA levels in C2C12 cells transfected with CaMKK2 plasmids (n = 3); (**B**) Representative western blot showing the CaMKK2 protein levels in C2C12 cells transfected with CaMKK2 plasmids; (**C**) Representative western blot showing the cleaved caspase3 and Bcl-xL protein levels in C2C12 cells transfected with CaMKK2 plasmids; (**D**) CaMKK2 mRNA levels in C2C12 cells transfected with siCaMKK2 (n = 3); (**E**) Representative western blot showing the CaMKK2 protein levels in C2C12 cells transfected with siCaMKK2; (**F**) Representative micrographs of crystal violet-stained C2C12 cell colonies, which were transfected with siCaMKK2-b as indicated (**left**), absorbance of each well at 590 nm (**right**) (n = 3); (**G**) Representative western blot showing the CaMKK2 and p-Cdc2 Tyr15 protein levels in C2C12 cells transfected with siCaMKK2-b; (**H**) Representative western blot showing the MYH, MEF2c protein levels in C2C12 myotubes transfected with siCaMKK2-b; (**I**) Immunofluorescence analysis of MYH was performed in C2C12 myotubes 48 h after siCaMKK2-b transfection (**left**), quantification of the fusion index of indicated cells (**right**) (n = 3); (**J**) Representative western blot showing the p-AMPK and CaMKK2 protein levels in undifferentiated (Day 0, 0d) and differentiated (Day 2, 2d) C2C12 cells as indicated. Experiments were repeated at least twice. Means ± SEM (error bars) are shown. *, $p < 0.05$; **, $p < 0.01$, ***, $p < 0.001$.

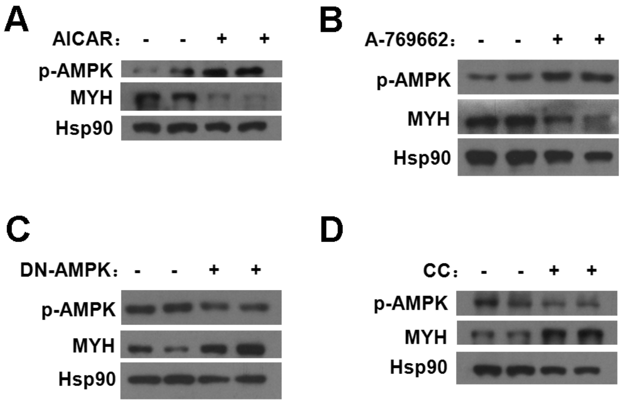

Figure S3. AMPK activation or inhibition affectsC2C12 myoblasts differentiation. (**A**,**B**) Representative western blot showing the MYH and p-AMPK protein levels in C2C12 myotubes treated with 0.25 mM AICAR or 80 μM A-769662 (**B**) for 48 h; (**C**,**D**) Representative western blot showing the MYH and p-AMPK protein levels in C2C12 myotubes treated with DN-AMPK (**C**) or10 μM Compound **C** (D, CC) for 48 h. Experiments were repeated at least twice.

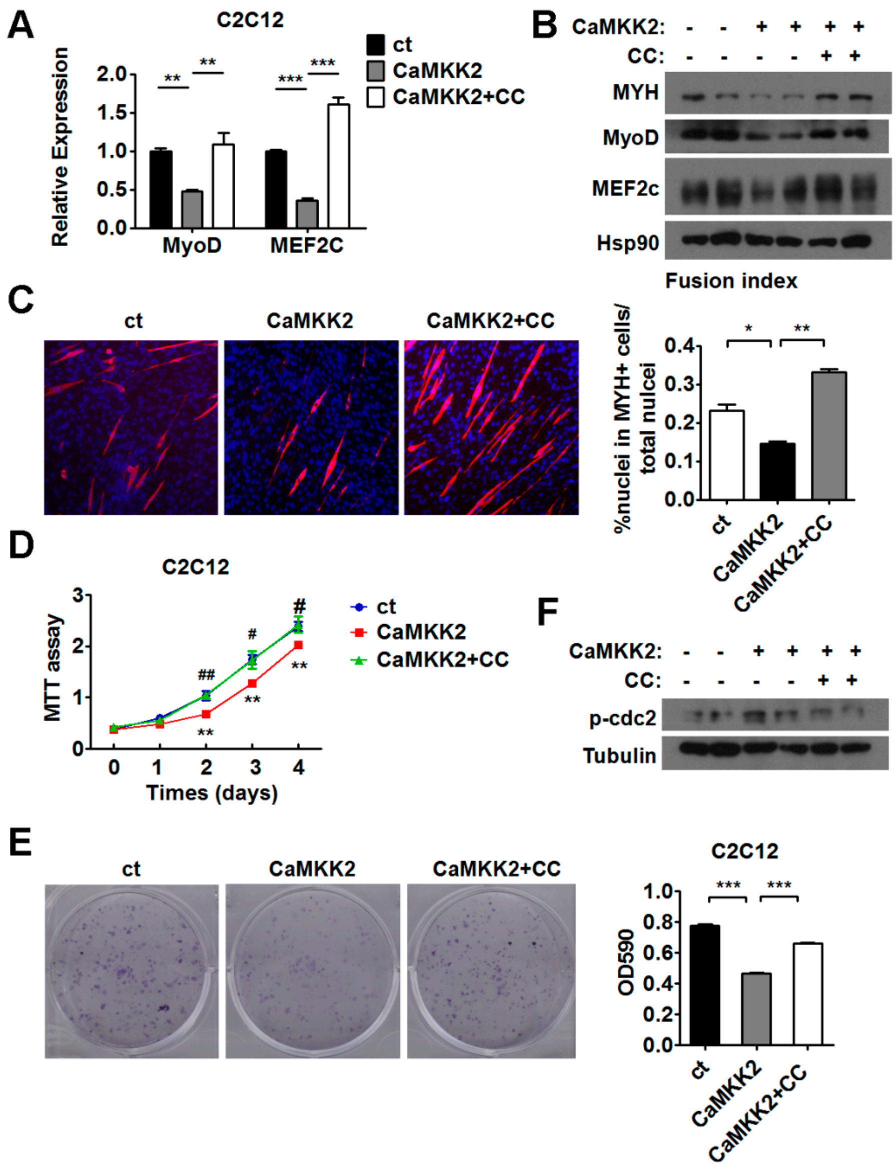

Figure S4. CaMKK2 inhibits C2C12 myoblasts proliferation and differentiation through AMPK activation. (**A**) Quantitative RT-PCR analysis of MyoD and MEF2c mRNA levels in C2C12 myotubes transfected with CaMKK2 plasmids and then treated with 10 μM Compound C (CC) for 48 h (n = 3); (**B**) Representative western blot showing the MYH, MyoD and MEF2c protein levels in C2C12 myotubes transfected with CaMKK2 plasmids and then treated with 10 μM Compound C for 48 h; (**C**) Immunofluorescence analysis of MYH was performed in C2C12 myotubes transfected with CaMKK2 plasmids and then treated with 10 μM Compound C for 48 h (**left**), quantification of the fusion index of indicated cells (right) (n = 3). Means ± SEM (error bars) are shown. *, $p < 0.05$; **, $p < 0.01$; ***, $p < 0.001$; (**D**) MTT assay of the proliferation ability of C2C12 myoblasts transfected with CaMKK2 or control plasmids, and then incubated with 10 μM Compound C and maintained over a period of 5 days. Means ± SEM (error bars) are shown. **, $p < 0.01$ CaMKK2-treated group vs. control group; #, $p < 0.05$; ##, $p < 0.01$ CaMKK2 and CC treated group vs. CaMKK2-treated group; (**E**) Representative micrographs of crystal violet-stained C2C12 cell colonies, which were transfected with CaMKK2 or control plasmids, and then incubated with 10 μM Compound C (**left**), absorbance of each well at 590 nm (**right**) (n = 3); (**F**) Representative western blot showing the p-cdc2 protein levels in C2C12 myotubes transfected with CaMKK2 plasmids and then treated with 10 μM Compound C for 48 h. Experiments were repeated at least twice. Means ± SEM (error bars) are shown. ***, $p < 0.001$.

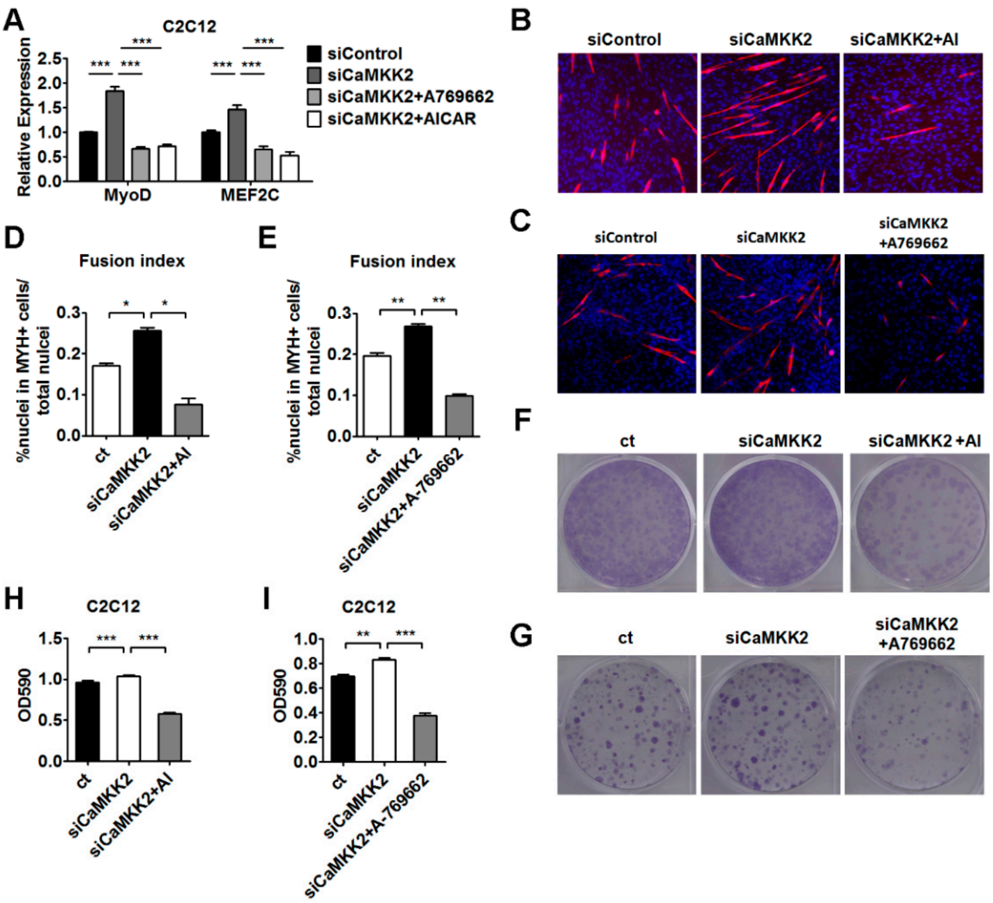

Figure S5. A-769662 and AICAR attenuates the effect of CaMKK2 knockdown in C2C12 myoblasts proliferation and differentiation. (**A**) Quantitative RT-PCR analysis of MyoD, and MEF2c mRNA levels in C2C12 myotubes transfected with siCaMKK2 and then treated with 0.25 mM AICAR or 80 μM A-769662 for 48 h ($n = 3$); (**B**,**C**) Immunofluorescence analysis of MYH was performed in C2C12 myotubes transfected with siCaMKK2 and then treated with 0.25 mM AICAR (**B**) or 80 μM A-769662 (**C**) for 48 h; (**D**,**E**) Quantification of the fusion index of C2C12 myotubes transfected with siCaMKK2 and then treated with 0.25 mM AICAR (**D**) or 80 μM A-769662 (**E**) for 48 h ($n = 3$); (**F**,**G**) Representative micrographs of crystal violet-stained C2C12 cell colonies, which were transfected with siCaMKK2 and then treated with 0.25 mM AICAR (**F**) or 80 μM A-769662 (**G**) for 48 h; (**H**,**I**) Absorbance at 590 nm of crystal violet-stained C2C12 cell colonies, which were transfected with siCaMKK2 and then treated with 0.25 mM AICAR (**H**) or 80 μM A-769662 (**I**) for 48 h ($n = 3$). Means ± SEM (error bars) are shown. *, $p < 0.05$; **, $p < 0.01$; ***, $p < 0.001$.

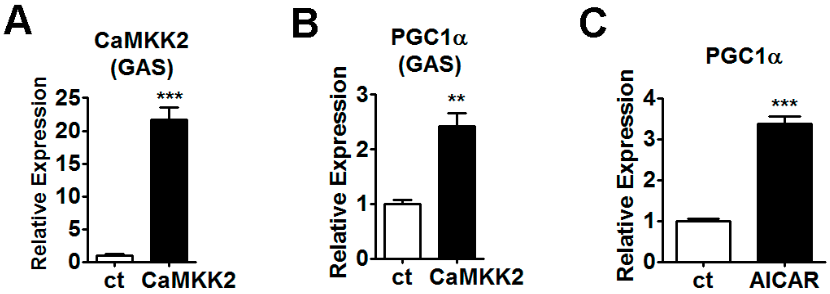

Figure S6. CaMKK2 expression in mice after electroporation with CaMKK2 plasmids and PGC1*α* expression in C2C12 cells treated with AICAR. (**A**,**B**) Quantitative RT-PCR analysis of CaMKK2 (**A**) and PGC1*α* (**B**) mRNA levels in gastrocnemius muscles (GAS) after electroporation with CaMKK2 and control plasmids ($n = 3$); (**C**) Quantitative RT-PCR analysis of PGC1*α* mRNA levels in C2C12 cells treated with 0.25 mM AICAR for 48 h ($n = 3$). Means ± SEM (error bars) are shown. **, $p < 0.01$; ***, $p < 0.001$.

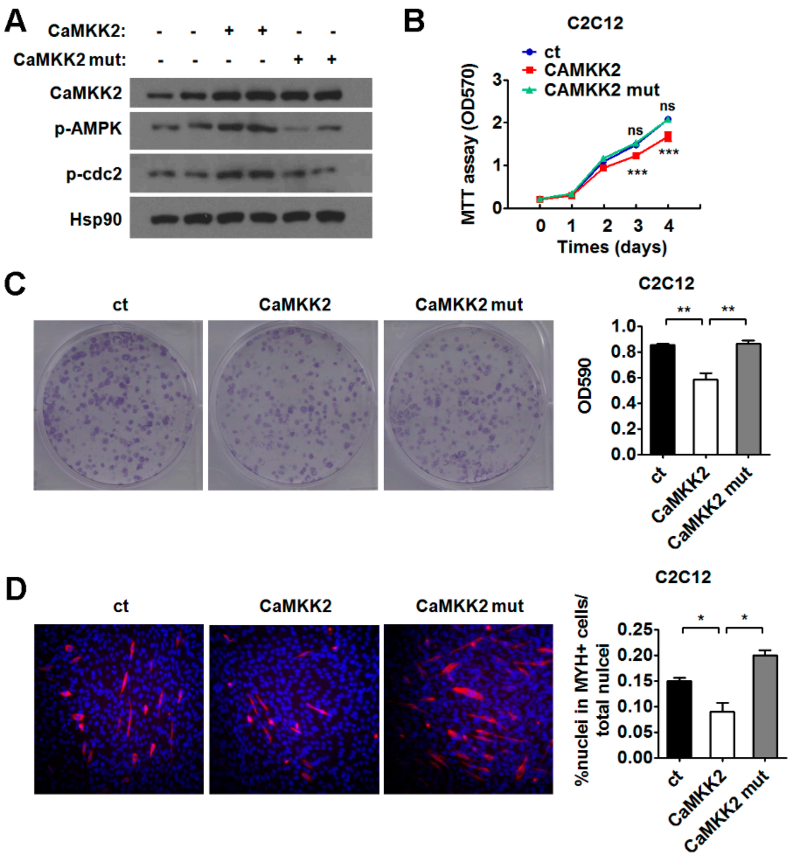

Figure S7. CaMKK2 K193A mutation could not lead to cell cycle retardation or inhibit myoblast differentiation in vitro. (**A**) Representative western blot showing the p-AMPK and p-cdc2 protein levels in the C2C12 cells transfected with CaMKK2 or CaMKK2 K193A plasmids; (**B**) MTT assay of the proliferation of C2C12 myoblasts transfected with CaMKK2 or CaMKK2 K193A plasmids and maintained over a period of 5 days. Means ± SEM (error bars) are shown. ***, $p < 0.001$, CaMKK2-treated group vs. control group; ns, $p \geq 0.05$ CaMKK2-mut treated group vs. CaMKK2-treated group; (**C**) Representative micrographs of crystal violet-stained C2C12 cell colonies, which were transfected with CaMKK2 or CaMKK2 K193A plasmids as indicated (**left**), absorbance of each well at 590 nm (**right**) ($n = 3$); (**D**) Immunofluorescence analysis of MYH was performed in C2C12 myotubes 48 h after CaMKK2 plasmids transfection (**left**), quantification of the fusion index of indicated cells (**right**) ($n = 3$). Experiments were repeated at least twice. Means ± SEM (error bars) are shown. *, $p < 0.05$; **, $p < 0.01$.

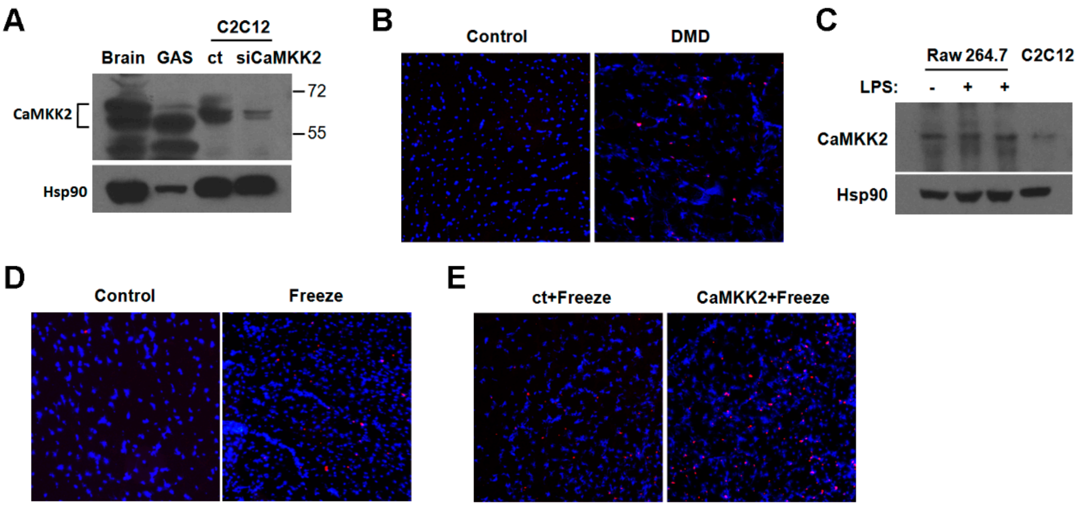

Figure S8. CD68 staining in DMD patient and mice gastrocnemius muscles. (**A**) Representative western blot showing the CaMKK2 protein levels in brain, gastrocnemius muscle and C2C12 cell transfected with siCaMKK2 or ct; (**B**) CD68 staining of muscle from DMD patient (Blue: DAPI, Red: CD68); (**C**) Representative western blot showing the CaMKK2 protein levels in Raw 264.7 and C2C12 cells; (**D**) CD68 staining of regenerating gastrocnemius muscle (**Blue**: DAPI, **Red**: CD68); (**E**) CD68 staining of regenerating gastrocnemius muscles from mice after electroporation with CaMKK2 and control plasmids at 14 days following freeze injury (**Blue**: DAPI, **Red**: CD68). Experiments were repeated at least twice.

Table S1. Primers for quantitative real-time PCR.

Gene	Accession No.	Primer Sequence	Product Length
CaMKK2	NM_001199676	Forward: AGACCAGGCCCGCTTCTACT<br>Reverse: GAAGATCTTGCGGGTCTCTG	241
MyoD	NM_010866.2	Forward: CGCCACTCCGGGACATAG<br>Reverse: GAAGTCGTCTGCTGTCTCAAAGG	71
MEF2C	NM_001170537.1	Forward: GTCAGTTGGGAGCTTGCACTA<br>Reverse: CGGTCTCTAGGAGGAGAAACA	112
Myogenin	NM_031189.2	Forward: AGTGAATGCAACTCCCACAG<br>Reverse: ACGATGGACGTAAGGGAGTG	137
cyclin A1	NM_001305221.1	Forward: TGATGCTTGTCAAATGCTCAGC<br>Reverse: AGGTCCTCCTGTACTGCTCAT	101
cyclin D1	NM_007631.2	Forward: GCGTACCCTGACACCAATCTC<br>Reverse: CTCCTCTTCGCACTTCTGCTC	183
cyclin E1	NM_007633.2	Forward: GTGGCTCCGACCTTTCAGTC<br>Reverse: CACAGTCTTGTCAATCTTGGCA	101
p27	NM_009875.4	Forward: TCAAACGTGAGAGTGTCTAACG<br>Reverse: CCGGGCCGAAGAGATTTCTG	103
Pax7	NM_011039.2	Forward: TCTCCAAGATTCTGTGCCGAT<br>Reverse: CGGGGTTCTCTCTCTTATACTCC	132
PGC1α	NM_008904.2	Forward: CAATGAATGCAGCGGTCTTA<br>Reverse: GTGTGAGGAGGGTCATCGTT	198
GAPDH	NM_001289726.1	Forward: ACATCATCCCTGCATCCACT<br>Reverse: GTCCTCAGTGTAGCCCAAG	224

Table S2. Densitometric analysis for Western blots.

Figure 1									
	**Figure 1B**	**1**	**2**	**3**	**4**	**5**	**6**	**7**	**8**
	1	1.00	1.52	1.17	1.20	0.39	0.60	0.22	0.38
	2	1.00	0.93	1.46	1.37	1.18	1.18	1.25	1.76
	**Figure 1C**	**1**	**2**	**3**	**4**	**5**	**6**		
	1	1.00	0.2	0.82	0.49	0.80	0.49		
	2	1.00	1.19	0.91	1.02	1.14	1.40		
	**Figure 1D**	**1**	**2**	**3**	**4**	**5**	**6**		
	1	1.00	0.12	0.84	0.86	1.47	0.67		
	2	1.00	1.15	1.05	1.43	1.07	1.03		
	**Figure 1G**	**1**	**2**	**3**	**4**	**5**	**6**		
	1	1.00	2.22	5.85	11.43	16.90	20.05		
	2	1.00	2.55	4.15	4.58	5.24	4.31		
	3	1.0	0.90	0.79	0.80	0.64	0.59		
	4	1.00	1.23	0.83	0.66	0.45	0.22		
	5	1.00	0.99	0.96	0.93	0.94	1.06		
Figure 2									
	**Figure 2F**	**1**	**2**	**3**	**4**				
	1	1.00	1.33	2.43	2.76				
	2	1.00	1.06	1.04	1.14				
	**Figure 2H**	**1**	**2**	**3**	**4**				
	1	1.00	1.89	2.92	3.45				
	2	1.00	0.86	0.52	0.29				
	3	1.00	0.97	0.44	0.40				
	4	1.00	0.74	0.84	0.80				
Figure 3									
	**Figure 3B**	**1**	**2**	**3**	**4**				
	1	1.00	1.13	1.81	1.42				
	2	1.00	0.89	0.64	0.36				
	3	1.00	0.90	0.58	0.56				
	4	1.00	0.83	0.37	0.55				
	5	1.00	1.07	1.10	1.00				
Figure 4									
	**Figure 4E**	**1**	**2**	**3**	**4**				
	1	1.00	0.79	0.55	0.39				
	2	1.00	0.94	0.60	0.71				
	3	1.00	1.21	0.68	0.67				
	4	1.00	0.89	0.97	0.94				
	**Figure 4H**	**1**	**2**	**3**	**4**				
	1	1.00	1.07	0.70	0.57				
	2	1.00	1.19	1.92	1.82				
	3	1.00	1.34	2.49	2.26				
	4	1.00	1.02	0.85	0.82				

Table S2. *Cont.*

Figure 5							
	**Figure 5A**	**1**	**2**	**3**	**4**		
	1	1.00	1.08	1.68	1.83		
	2	1.00	1.20	1.81	1.77		
	3	1.00	0.80	0.83	0.80		
	4	1.00	1.11	1.04	0.92		
	**Figure 5C**	**1**	**2**	**3**	**4**	**5**	**6**
	1	1.00	0.82	0.35	0.63	1.37	1.28
	2	1.00	1.05	0.83	0.64	1.05	1.12
	3	1.00	1.03	0.70	0.47	0.74	0.89
	4	1.00	0.87	0.87	0.96	0.79	0.85
	5	1.00	1.03	1.73	1.89	0.97	0.58
	6	1.00	0.76	1.05	1.07	1.82	1.74
	**Figure 5G**	**1**	**2**	**3**	**4**	**5**	**6**
	1	1.00	1.13	1.57	1.31	1.05	1.10
	2	1.00	1.26	1.19	0.95	1.01	0.93
Figure 6							
	**Figure 6B**	**1**	**2**	**3**	**4**		
	1	1.00	1.07	1.89	1.51		
	2	1.00	1.14	1.12	1.01		
	**Figure 6D**	**1**	**2**	**3**	**4**		
	1	1.00	0.76	0.25	0.47		
	2	1.00	1.20	1.13	1.23		
	**Figure 6G**	**1**	**2**	**3**	**4**		
	1	1.00	1.04	3.59	3.61		
	2	1.00	1.10	0.86	0.97		
	**Figure 6H**	**1**	**2**	**3**	**4**		
	1	1.00	0.96	1.72	1.71		
	2	1.00	1.02	0.85	0.87		
